# Characteristics of Microaneurysm Size in Residual Edema After Intravitreal Injection of Faricimab for Diabetic Macular Edema

**DOI:** 10.3390/jcm13247839

**Published:** 2024-12-22

**Authors:** Yutaka Yamada, Yoshihiro Takamura, Masakazu Morioka, Hideyuki Oshima, Makoto Gozawa, Takehiro Matsumura, Masaru Inatani

**Affiliations:** Department of Ophthalmology, Faculty of Medical Sciences, University of Fukui, Eiheiji-cho, Yoshida-gun, Fukui-ken 910-1193, Fukui, Japan; yyutaka@u-fukui.ac.jp (Y.Y.); mmorioka@u-fukui.ac.jp (M.M.); osm@g.u-fukui.ac.jp (H.O.); makotogozawa@gmail.com (M.G.); tmatsu@u-fukui.ac.jp (T.M.); inatani@u-fukui.ac.jp (M.I.)

**Keywords:** diabetic macula edema, faricimab, microaneurysm, DME, VEGF, Ang-2

## Abstract

**Background/Objectives:** Microaneurysms (MAs) are important in the pathology of diabetic macular edema (DME) and its response to anti-vascular endothelial growth factor (VEGF) therapy. This study aimed to clarify the morphological characteristics of MAs in residual edema following consecutive faricimab injections, a bispecific antibody against angiopoietin-2 and VEGF. **Methods:** We selected patients with DME who exhibited residual edema after three monthly injections of faricimab. In both the residual and absorbed areas of edema, we counted the turnover of MAs, including those that were lost and those that were newly formed. The total number of MAs was determined based on the merged images from an optical coherence tomography (OCT) map and fluorescein angiography. **Results:** A total of 8 of the 42 patients who received three monthly injections of faricimab showed residual edema one month after the injections. In the residual edema, the density of MAs and the number of maintained MAs were significantly higher (*p* = 0.04), while the number of disappeared MAs (*p* = 0.04) and MA turnover (*p* = 0.01) were lower compared to the absorbed areas. Among the MAs that persisted after the initial injection, the proportion of large-sized MAs (*p* = 0.01) and their density were significantly greater than those in the absorbed area. In conclusion, the residual areas following three doses of faricimab displayed a higher MA density, less MA loss, and a high density of large-sized MAs compared to the absorbed areas. Our data suggest that large-size MAs located in the residual edema are characteristic of DME cases refractory to faricimab treatment.

## 1. Introduction

Diabetic macular edema (DME) is the leading cause of central vision loss in individuals with diabetes mellitus (DM) and can develop at any stage of diabetic retinopathy (DR) [1]. Prolonged hyperglycemia leads to hypoxia and chronic micro-vascular damage to the retina. Currently, intravitreal injections of anti-vascular endothelial growth factor (VEGF) agents are the first-line treatment for DME [2]. Numerous clinical studies have demonstrated that repeated intravitreal injections of anti-VEGF agents significantly improve visual acuity and reduce retinal thickness [3,4,5]. However, approximately 40% of cases remain refractory and exhibit a poor response to this treatment [6].

In refractory DME, residual focal edema is occasionally observed even after repeated anti-VEGF injections. Following the injection of the anti-VEGF agent, the improvement ratio of the retinal thickness of the residual edema is low compared to the areas that the edema have absorbed, suggesting a poor response to anti-VEGF therapy [7]. In the regions of residual edema after treatment, high-density microaneurysms (MAs) are present both before and after injections [7,8]. According to a report by Lee et al., cases with a poor response to anti-VEGF therapy are characterized by low vascular flow density in the deep capillary plexuses and a high number of adjacent MAs [9]. These findings indicate that high-density MAs may be a risk factor for resistance to anti-VEGF therapy in DME.

Four types of VEGF inhibitors have been approved for treating DME in Japan: ranibizumab, aflibercept, brolucizumab, and faricimab. Ranibizumab and brolucizumab specifically inhibit VEGF-A, while aflibercept inhibits VEGF-A, VEGF-B, and placental growth factor. Faricimab is a bispecific antibody that simultaneously targets VEGF-A and angiopoietin-2 (Ang)-2 [10]. Recently, we demonstrated that the intravitreal injection of faricimab reduced the number of MAs in patients with DME by approximately 60–70% [11]. A merged analysis of fluorescein angiography (FA) images taken before and after treatment revealed that faricimab injections inhibited the new onset of MAs and reduced the total number of existing MAs.

Nevertheless, several cases of refractory DME show residual edema after repeated injections of faricimab. To address refractory DME, it is important to clarify the characteristics of MAs that persist after faricimab injection. In this study, we analyzed the morphological characteristics of MAs present in residual edema, even after three consecutive injections of faricimab.

## 2. Materials and Methods

This study includes a secondary analysis of our previous work [11]. We conducted a retrospective analysis of 42 eyes from 42 consecutive patients diagnosed with type 2 DM and DME. Our protocol adhered to the principles of the Declaration of Helsinki and received approval from the Institutional Review Board of the University of Fukui. Informed consent was obtained from all patients after this study’s intent was thoroughly explained. Following three monthly injections of faricimab, we selected patients who exhibited residual edema one month later and categorized them as “residual edema cases”. Residual edema was defined as areas of high thickness (>500 μm) displayed as white regions on an optical coherence tomography (OCT) map.

### 2.1. Inclusion and Exclusion Criteria

The inclusion criteria were as follows: (1) age greater than 20 years; (2) a diagnosis of type 2 DM with DME; and (3) the use of faricimab as anti-VEGF therapy for diffuse DME with a central retinal thickness (CRT) exceeding 300 µm in one eye. The exclusion criteria included the following: (1) a history of the of use of anti-VEGF drugs or steroid injections or retinal photocoagulation within six months before the initial injection of faricimab; (2) active intraocular inflammation or infection; (3) uncontrolled glaucoma in either eye; (4) other retinal diseases, such as retinal vein occlusion or retinal detachment; (5) history of stroke; (6) systolic blood pressure (BP) exceeding 180 mmHg, diastolic BP exceeding 100 mmHg, or untreated hypertension; and (7) severe medial opacity that precluded fundoscopic evaluation (for example, severe cataract, corneal opacity, or vitreous hemorrhage). In this study, we enrolled patients with diffuse and center-involved DME treated with faricimab but excluded those with focal DME. Diffuse DME was defined as (1) increased retinal thickness with center involvement on an OCT map and (2) fluorescein leakage beginning in the early phase and continuing to increase into the late phase.

### 2.2. Procedures

All patients underwent comprehensive ophthalmic examinations during their initial visit, which included best-corrected visual acuity (BCVA) testing, slit-lamp microscopy, intraocular pressure measurement, and fundoscopy. BCVA values were converted to the logarithm of the minimum angle of resolution (logMAR) scale. Color fundus photographs were captured using a Kowa VX-10i fundus camera (Kowa Ltd., Nagoya, Japan), while the images of FA and the OCT map, including the CRT measurement, were obtained using a Spectralis Heidelberg Retinal Angiography (Heidelberg Engineering, Heidelberg, Germany). Using OCT, we obtained automatically reconstructed false-color topographic images that displayed the average thickness in each of the nine map sectors, as defined by the Early Treatment Diabetic Retinopathy Study criteria, to assess retinal thickness. The scanned areas measured 6 × 6 mm^2^, with the fovea at the center. Hyperfluorescent dots were detected during the early phase of FA imaging (within one min after dye injection). All imaging tests were performed by an experienced ophthalmologist who was blinded to the treatment status.

Intravitreal injections were administered in a standardized manner by a trained ophthalmologist (Y.Y.), utilizing 0.4% oxybuprocaine hydrochloride (0.4% benoxyl ophthalmic solution; Santen Co., Ltd., Osaka, Japan) and 2% xylocaine as anesthetics, along with povidone-iodine as an antiseptic. An eyelid speculum was used to stabilize the eyelids during the procedure. The injection volume of faricimab (VABYSMO; Cyugai Pharmaceutical Co., Ltd., Tokyo, Japan) was 2 mg/0.05 mL. Three injections were administered monthly. An examination was performed before the injection on the day of the first dose (day 0) and one and two months after the second and third injections, followed by another examination four weeks later (three months post-injection).

### 2.3. Merged Imaging Technique to Analyze MA Turnover

As previously described, we merged the FA images taken before and after treatment to analyze MA turnover using Adobe Photoshop Elements (Adobe Systems Inc., San Jose, CA, USA) [11]. Initially, we marked the MAs in the FA images from day 0 (before treatment) in red and week 12 (after treatment) in green. The transparency of each image was reduced by 50%, allowing us to overlap the images with retinal vessels. When the red MA overlaps the green MA, it becomes a yellow MA, meaning it was persistently present before and after treatment. We counted the numbers of red, green, and yellow MAs, representing disappeared, newly developed, and maintained MAs within a 6 × 6 mm^2^ area. MA turnover was defined as the sum of newly developed and disappearing MAs. The FA images were merged with the simultaneously captured OCT map. The numbers of lost, newly generated, and maintained MAs were expressed as percentages, with the number of MAs before injection serving as the baseline (100%). The reduction rate of MAs was calculated by dividing the difference in MAs before and after treatment by the number of MAs before treatment, which was also expressed as a percentage. MAs were categorized into three groups based on their diameters: small (less than 3 pixels), medium (3–7 pixels), and large (>7 pixels). The diameter of MAs was converted from pixels to μm based on the 200 μm scale displayed on the FA images.

### 2.4. Statistical Analysis

Statistical analyses were performed using JMP software version 13.0 (SAS Institute Inc., Tokyo, Japan). Variables were expressed as the means ± standard deviation (SD). We used Bartlett’s test to examine the equality of variance across the samples. After confirming the normal distribution of the data, the number of MAs was compared between time points using the Wilcoxon signed-rank test. Differences between the residual and absorbed areas were analyzed using the Mann–Whitney U test. Statistical significance was set at *p* < 0.05.

## 3. Results

**Figure 1:**
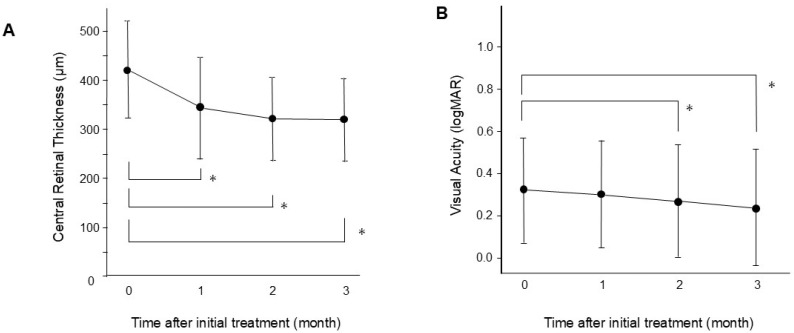
Changes in central retinal thickness (**A**) and best-corrected visual acuity (BCVA) (**B**) eyes treated with faricimab injections. BCVA is expressed as logMAR. Data are presented as mean ± standard deviation. * *p* < 0.05 (versus baseline).

Faricimab was administered via injection three times per month in 42 patients with DME. One month later, eight patients exhibited residual edema. Three of these eight patients presented center-involved DME, while the other five had non-center-involved focal DME at the three-month follow-up. Table 1 displays the baseline characteristics of the patients. None of the patients experienced adverse events following the injections, including retinal detachment, endophthalmitis, or vitreous hemorrhage. CRT significantly decreased at one month and continued to show improvement thereafter (*p* < 0.0001) (Figure 1). Furthermore, compared with baseline measurements, BCVA improved at two months (*p* = 0.03) and three months (*p* = 0.002).

**Figure 2:**
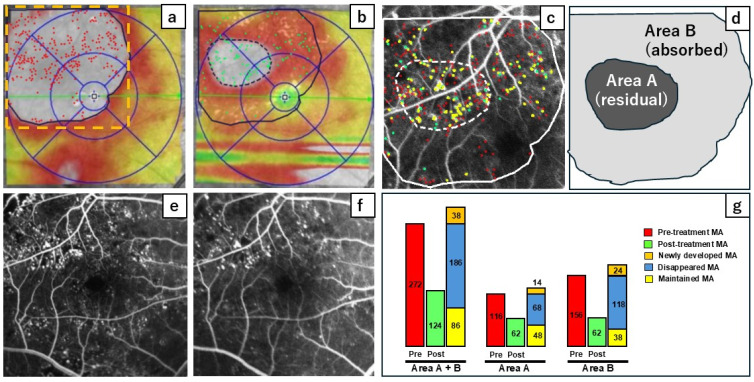
This case study illustrates the turnover microaneurysms (MAs) in an eye exhibiting residual edema following treatment with faricimab. A fluorescein angiography (FA) image was merged with an optical coherence tomography (OCT) map. The MAs observed before treatment (**a**) are marked in red, while those after treatment (**b**) are indicated in green. FA images taken before (**e**) and following (**f**) treatment were also merged (**c**). The residual and absorbed areas are defined as Areas A and B, respectively (**d**). (**g**) The accompanying color bar indicates the status of MAs: before treatment (red), after treatment (green), newly developed (orange), disappeared (blue), and maintained (yellow).

In the cases of residual edema, we analyzed changes in the number of MAs and their turnover in the area with residual edema (Area A) and where the edema had been absorbed (Area B). A representative example is illustrated in Figure 2. After treatment, the total number of MAs decreased from 272 (indicated by red dots in Figure 2a) to 124 (represented by green dots in Figure 2b). The number of MAs that disappeared (shown as red dots in Figure 2c) was 186, while the number of newly developed MAs (shown as green dots in Figure 2c) was 38. The count of retained MAs (depicted as yellow dots in Figure 2c) was 86. These analyses were performed in parallel for Areas A and B (Figure 2g).

**Figure 3:**
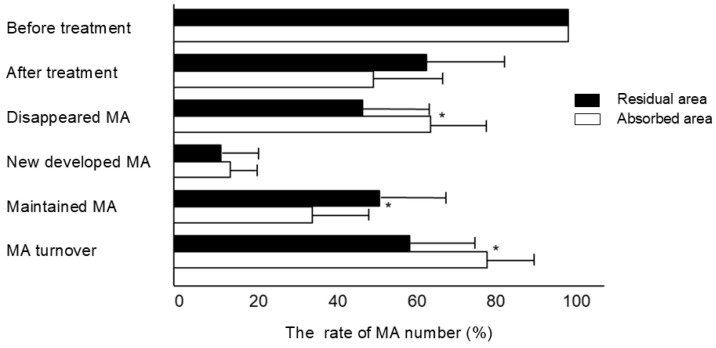
The rate of MA number in terms of total, disappeared, newly formed, maintained, and turnover was evaluated in the residual and absorbed areas after treatment with faricimab (* *p* < 0.05). The rate of MA number was calculated using MA counts before treatment as 100%.

As illustrated in Figure 3, the total number of MAs in the residual area decreased to 64.1 ± 19.9% compared to 50.6 ± 17.9% in the absorbed area, with before-treatment values considered to be 100%. The number of MAs that disappeared in the residual area (47.9 ± 16.3%) was significantly lower (*p* = 0.04) than that in the absorbed area (64.9 ± 14.2%). Conversely, there was no significant difference in the number of newly developed MAs between the two areas (residual area: 11.9 ± 9.1%, absorbed area: 14.3 ± 5.9%). Consequently, the turnover of MAs, the sum of new-onset and disappeared MAs, was significantly higher in the absorbed area (79.3 ± 12.3%) than that in the residual area (59.8 ± 17.3%) (*p* = 0.01). Furthermore, the number of maintained MAs in the residual area (52.1 ± 16.3%) was significantly higher than that in the absorbed area (35.1 ± 14.2%) (*p* = 0.04).

**Figure 4:**
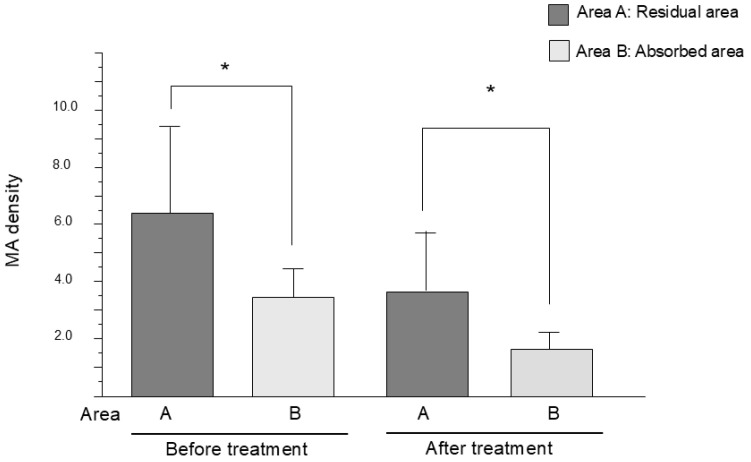
MA density in the residual area (Area A) and the absorbed area (Area B) before and after treatment with faricimab demonstrated a significant difference (* *p* < 0.05).

We measured the density of MAs localized in the edematous areas before and after treatment (see Figure 4). After treatment, the MA density in the residual edematous area (Area A: 3.81 ± 2.09) was significantly higher than that in the absorbed area (Area B: 1.65 ± 0.47) (*p* = 0.04). In the before-treatment phase, the MA density was higher in areas where focal edema was formed after treatment (6.39 ± 3.46) than that in areas that had absorbed (3.59 ± 1.05) (*p* = 0.01).

**Figure 5:**
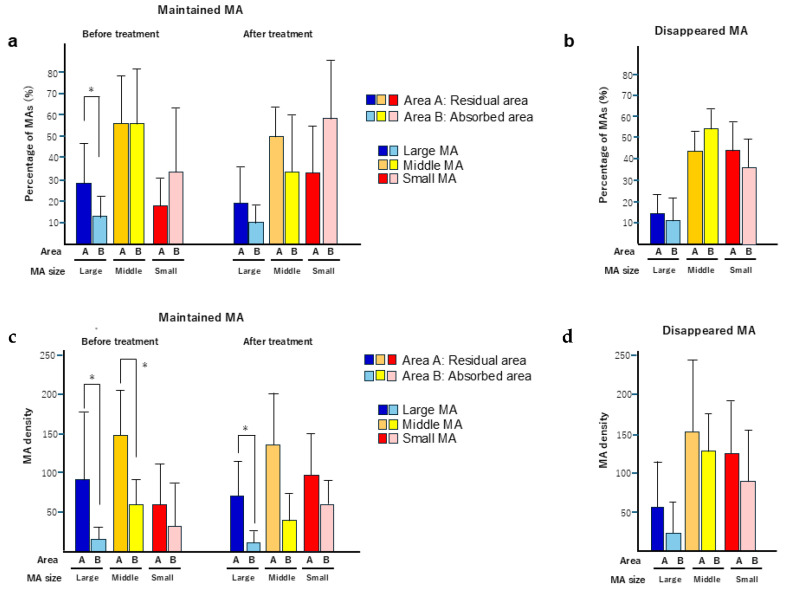
(**a**) The percentage of maintained MA was compared between the remaining and absorbed areas for each size (large, medium, and small). The graph on the left shows before treatment, and the right shows after treatment. (**b**) The percentage of disappeared MA was compared between the remaining and absorbed areas for each size (large, medium, and small). (**c**) The density of maintained MA was compared between the remaining and absorbed areas for each size (large, medium, and small). The graph on the left shows before treatment, and the right shows after treatment. * *p* < 0.05. (**d**) The density of disappeared MA was compared between the remaining and absorbed areas for each size (large, medium, and small).

Next, we analyzed the differences in the sizes of the MAs that persistently existed during the observation period between the residual edema and the absorbed area (Figure 5a). Before treatment, the proportion of large-sized MAs in the residual area was 27.6 ± 18.1%, significantly higher than that in the absorbed area (12.6 ± 9.2%; *p* = 0.01). We also examined the size distribution of MAs that disappeared after treatment in both the residual and absorbed areas. There was no significant difference in the proportion of each size in MAs between the residual area and the absorbed area (Figure 5b). The proportions of large MAs were only 15.4% in the residual area and 11.6% in the absorbed areas, respectively. We also showed the density of the large-, middle-, and small-size MAs in the residual and absorbed areas. In the maintained MAs, the density of the large MAs in the residual area was significantly higher than that in the absorbed areas before (*p* = 0.026) and after (*p* = 0.034) treatment, while the density of middle-size MAs was significantly higher before treatment (*p* = 0.01) (Figure 5c). No significant difference in the density of the disappeared MAs was noticed in each size of MA between the residual and absorbed areas (Figure 5d).

There was no significant relationship between the average size of MAs in the residual edema and CRT after faricimab treatment.

## 4. Discussion

Herein, we demonstrated that MAs are densely located in the areas of residual focal edema following the intravitreal injection of anti-VEGF drugs, including ranibizumab and aflibercept [8]. Although anti-VEGF therapy can improve center-involved DME to non-center-involved DME, residual edema often expands and reverts to center-involved DME due to ongoing leakage from MAs [12]. Therefore, there is a need for a new anti-VEGF drug that can effectively reduce MAs. Faricimab, a novel bispecific antibody targeting Ang-2 and VEGF-A, has been shown to reduce MAs by approximately 60–70%, compared to a 50% reduction with aflibercept [11,13,14]. However, even after three injections of faricimab, focal edema with high-density MAs persisted in 19.0% (8/42) of the patients. In the present study, we analyzed the differences in the dynamics of MAs between areas of residual and absorbed edema to explore the characteristics of MAs that remained after successive injections of faricimab.

The changes in the number of MAs are determined by increases due to the formation of MAs and decreases due to their disappearance. The sum of increased and disappeared MAs is recognized as MA turnover and serves as an indicator of DR and DME activity [15]. MAs are characterized by pericyte loss and the aberrant proliferation of endothelial cells (ECs) [16]. Faricimab has the potential to inhibit MA formation by simultaneously blocking VEGF and Ang-2, which are key players in the dynamics of pericytes and ECs. In normal vessels comprising pericytes and ECs, vascular stability and homeostasis are maintained through signaling via Ang-1 and the tyrosine-protein kinase receptor (Tie2) [17]. However, Ang-2 antagonizes Ang1/Tie2 signaling in pathological vessels, destabilizes the vessels, weakens junctions between ECs, and increases vascular leakage [17]. In pericytes experiencing apoptosis due to hyperglycemia, Ang-1 promotes cell survival, while Ang-2 exacerbates apoptosis [18]. Further, Park et al. demonstrated that Ang-2 induces apoptosis in pericytes through the p53 pathway under hyperglycemic conditions. In MAs with pericyte loss, there is an elevation in Ang-2 levels [19]. The eyes of Monkeys injected with VEGF exhibited MA formation, indicating a VEGF-mediated pathology process [20]. Thus, it is reasonable to conclude that the development of new MAs is inhibited by treatment with faricimab. The number of newly developed MAs was significantly lower in eyes treated with faricimab compared to the untreated eyes of the same patient [11]. In this study, the number of newly synthesized MAs was not a significant factor in forming residual edema due to its extremely low count. No significant difference was observed in the number of newly developed MAs between the absorbed and residual edema areas, suggesting that the formation of new MAs was strongly inhibited by faricimab treatment in both residual and absorbed areas.

In contrast to the onset of MAs, the disappearance of MAs in the residual edema was significantly lower than that in the absorbed area. The reason for the disappearance of MAs following faricimab injection remains unclear; however, faricimab may promote the recruitment of pericytes around retinal vascular ECs [21]. Alternatively, MAs that persist after faricimab administration have been shown to decrease in size after injection, possibly due to decreased perfusion within the MA. Zhang et al. reported that treatment with an anti-VEGF agent dynamically altered the morphology of MAs, with some patients exhibiting clearer wall reflexes, reduced contents, and diminished blood flow signals [22]. In this study, larger MAs were more prevalent in the residual regions. Moreover, the density of larger-size MAs in the residual area was higher than that in the absorbed area before and after faricimab treatment, indicating the involvement of the pathogenesis of residual edema formation. Dong et al. observed that large-sized MAs exhibited lumens that were partitioned into multiple chambers, lined by ECs only in certain areas, with the loss of pericytes [23]. It may be that the reduced number of cellular components, such as pericytes and ECs, which are targets for faricimab to act pharmacologically, contribute to the minimal reduction observed in larger MAs. Notably, it has been reported that the larger the MA before treatment, the fewer the number of leaked MAs [23]. However, even a small amount of persistent leakage may contribute to edema formation. Thus, in areas with numerous large MAs, faricimab may prove less effective, and both MAs and local edema may persist after treatment.

Recently, Castro-Farías et al. proposed the term ‘telangiectatic capillaries’ (TelCaps) for capillary anomalies larger than 150 μm, distinguishing them from “retinal arterial microaneurysms” [24]. The larger MAs in our study correspond to a diameter of approximately 70 µm or more, so they may include some TelCaps. Itou et al. reported that administering three consecutive injections of anti-VEGF agents could reduce the size of TelCaps in DME. Furthermore, the TelCaps that remained after treatment had a significantly larger mean size at baseline compared to those that resolved [25]. Indocyanine green molecules (IA) are 98% bound to proteins, preventing leakage through blood vessels [26]. Consequently, IA provides an enhanced visualization of TelCaps in DME [24]. However, since we did not employ IA in our retrospective study, we could not evaluate TelCaps.

Some limitations of this study, including its retrospective nature, are that only eight patients showed residual edema after treatment with faricimab, and the observational period was short. A long-term follow-up based on a larger sample size with fewer missing data and fewer lost follow-ups is needed. Moreover, refractory DME is not solely caused by unresolved MAs. Other biomarkers, such as hyper-reflective foci, DR severity, macular vessel architecture, and vessel density, can also influence treatment outcomes and should be further investigated.

In this study, we found that even after three doses of faricimab, the residual areas exhibited a higher MA density, less MA loss, and lower MA turnover than the absorbed areas. Additionally, large-sized MAs were more prevalent in residual edema areas and thus may be a risk factor in the formation of persistent edema following faricimab treatment. These findings suggest that large MAs may predict a poor response to anti-VEGF therapy and contribute to persistent edema. This study highlights the potential need for alternative therapies, such as direct photocoagulation aiming for large MAs in residual edema, to address resistant MAs and optimize treatment outcomes for refractory DME. However, it is important to understand that laser therapy is technically challenging and involves the thermal destruction of retinal tissue. Clinicians should carefully weigh the decision to continue repeated injections of anti-VEGF agents against the option of direct photocoagulation for MAs that are resistant to anti-VEGF therapy.

## Figures and Tables

**Table 1:** Baseline characteristics at time of registration.

Age (Years)	65.1 ± 8.8
Gender (male/female)	27/13
Duration of diabetes mellitus (years)	8.8 ± 7.5
Hemoglobin A l c (%)	7.5 ± 1.5
Serum creatinine	1.5 ± 2.0
Non-PDR:PDR	38:4
Number of MAs in edema	67.1 ± 37.8
Central retinal thickness (μm)	412.3 ± 98.4

PDR: proliferative diabetic retinopathy; MAs: microaneurysms.

## Data Availability

The data presented in this study are available on request from the corresponding author due to [specify the reason for the restriction].
